# The individualized selection of Pancreaticoenteric anastomosis in Pancreaticoduodenectomy

**DOI:** 10.1186/s12893-020-00791-y

**Published:** 2020-06-22

**Authors:** Ke-Min Jin, Wei Liu, Kun Wang, Quan Bao, Hong-Wei Wang, Bao-Cai Xing

**Affiliations:** Key Laboratory of Carcinogenesis and Translational Research (Ministry of Education/Beijing), Department of Hepatobiliary and Pancreatic Surgery Unit I, Peking University Cancer Hospital & Institute, No. 52, Fu-Cheng Road, Beijing, 100142 PR China

**Keywords:** Pancreaticoduodenectomy anastomosis POPF

## Abstract

**Background:**

The mortality following pancreaticoduodenectomy has markedly decreased but remains an important challenge for the complexity of operation and technical skills involved. The present study aimed to clarify the impact of individualized pancreaticoenteric anastomosis and management to postoperative pancreatic fistula.

**Methods:**

Data from 529 consecutive pancreaticoduodenectomies were retrospectively analysed from the Hepatobiliary and Pancreatic Surgery Unit I, Peking Cancer Hospital. The pancreaticoenteric anastomosis was determined based on the pancreatic texture and diameter of the main pancreatic duct. The amylase value of the drainage fluid was dynamically monitored postoperatively on days 3, 5 and 7. A low speed intermittent irrigation was performed in selected patients. Intraoperative and postoperative results were collected and compared between the pancreaticogastrostomy (PG) group and pancreaticojejunostomy (PJ) group.

**Results:**

From 2010 to 2019, 529 consecutive patients underwent pancreaticoduodenectomy. Pancreaticogastrostomy was performed in 364 patients; pancreaticojejunostomy was performed in 150 patients respectively. The clinically relevant pancreatic fistula (CR-POPF) was 9.8% and mortality was zero. The soft pancreas, diameter of main pancreatic duct≤3 mm, BMI ≥ 25, operation time > 330 min and pancreaticogastrostomy was correlated with postoperative pancreatic fistula significantly. The CR-POPF of PJ was significantly higher than that of PG in soft pancreas patients; the operation time of PJ was shorter than that of PG significantly in hard pancreas patients. Intraoperative blood loss and operation time of PG was less than that of PJ significantly in normal pancreatic duct patients (*p* < 0.05).

**Conclusions:**

Individualized pancreaticoenteric anastomosis should be determined based on the pancreatic texture and pancreatic duct diameter. The appropriate anastomosis and postoperative management could prevent mortality.

## Background

Pancreaticoduodenectomy (PD) remains the golden standard for periampullary cancers [1]. Due to the complexity of the procedures and postoperative life-threatening complications, the mortality rates still was 1.4–29% [2, 3]. Despite a significant improvement in postoperative outcomes during recent decades, only a limited number of reports have documented zero mortality in consecutive pancreaticoduodenectomy series.

Postoperative pancreatic fistula (POPF) is one of the most potentially fatal complication after PD with rate ranging from 40 to 70%, which might cause arterial bleeding and mortality rate to 11–60% [4–6]. The established risk factors for POPF included a small pancreatic duct size, a soft pancreas and its posterior location. There have been a number of reported managements to reduce the incidence of POPF. The approach to management of the pancreatic remnant and form of pancreaticoenteric anastomosis (PA) determined the chance of developing POPF. Many efforts have been made to improve technical considerations through various modifications of pancreaticojejunostomy (PJ) and reconstruction with pancreaticogastrostomy (PG). The prompt management of POPF also decreased mortality, included in prophylactic use of octreotide and antibiotics.

Despite numerous trials comparing diverse PA techniques and other adjunctive strategies (pancreatic duct stenting, somatostatin analogues, etc), currently, there is no clear consensus regarding the ideal method of PA. The present study aimed to verify our management of individualized PA during operation and intermittent irrigation.

## Methods

### Study population

Between 1st November 2010 and 30th December 2019, 529 consecutive PDs were performed in the Hepatobiliary and Pancreatic Surgery Unit I of Peking Cancer Hospital. No 90-day mortality was reported in any of the 529 patients. Preoperative data were recorded, including demographics, comorbidity, performance status, American Society of Anaesthesiologists (ASA) level, previous abdominal history, preoperative serous bilirubin level and preoperative biliary drainage. The cases included 52 benign diseases (9.83%) and 477 malignancies (90.17%).

### Surgical techniques

The texture of pancreatic parenchyma and the diameter of main pancreatic duct were assessed intraoperatively, which determined method of individualized PA. The texture was recorded as soft if elasticity of the pancreas was preserved (the stiffness of the patient’s forehead as the reference as mentioned before). Additionally, the diameter of the main pancreatic duct was measured at the surgically transected surface of the pancreas. PG was performed in patients with a normal main pancreatic duct (≤3 mm) and/or soft pancreas texture because the occurrence of clinically significant postoperative pancreatic fistula is more likely in such patients. It was performed with a two-layer purse-string suture on the posterior gastric wall, and the pancreas was mobilized 2-3 cm to be telescoped into the gastric cavity. PJ was performed in patients with the main duct was larger than 3 mm and hard pancreatic texture. It was performed with end-to-side two-layer sutures. The first layer was a duct-to-mucosa anastomosis, and the second-layer suture was located between the capsule of the pancreas and the seromuscular layer of the jejunum. A pancreatic duct stent was routinely used in all pancreaticodigestive reconstructions for internal drainage. For adequate drainage, two 20F Robinson drains were placed in addition to the pancreaticodigestive anastomosis.

### Postoperative management

The amylase level of the drainage fluid was routinely evaluated on postoperative days 3, 5 and 7. POPF was defined as any measurable drainage on postoperative day 3 with an amylase content greater than 3 times the upper limit of the normal serum amylase level, and the severity was also graded as A, B, or C according to the criteria proposed by the International Study Group on Pancreatic Fistula^6^. If the fluid was transparent with normal amylase level, the drains were removed as soon as possible, typically on postoperative days 8. The drains were maintained for longer if patients had high drain amylase activity, copious fluid output or effluent with an unfavorable appearance (dark brown, greenish, milky or murky). Antibiotics were selected based on the susceptibility of bacteria isolated from drain fluids. At meanwhile, an irrigating tube was placed along the drainage tube and a low-speed intermittent irrigation was performed until the drain fluid returned transparent, which intended to dilute concentrated amylase. The speed of the irrigating saline should be controlled to avoid it spread into uninvolved areas of the abdominal cavity. When intra-abdominal collection and associated symptoms were detected, ultrasound-guided percutaneous drainage was performed. Irrigation was stopped, and the drainage was removed when the daily collections decreased < 30 mL/24 h. Postoperative complications were graded by the Clavien-Dindo classification [7].

### Statistical analysis

Continuous variables were summarized as the medians and ranges, and categorical variables were summarized as frequencies and percentages. The Mann-Whitney U test was used to compare the continuous variables, and the Chi-square test was used to compare the categorical variables. A *p* value of < 0.05 was deemed statistically significant.

## Results

### Patient characteristics and surgical details

The demographic and comorbidity data were shown in Table 1. The median age of all patients was 61 years old (range: 18–82). Two hundred and ninety-four patients (55.6%) were male. The most common disease was pancreatic ductal adenocarcinoma (130 cases, 24.6%) (Supplementary Table 1). The median operation time was 287 min (range: 150-648 min), and the median intraoperative blood loss was 200 ml (range: 50-1500 ml). The median postoperative length of the hospital stay was 19 days (range: 8-121 days) (Table 2).

**Table 1:** Demographic and comorbidity data

	Number(%)/median (range)
**Age (years)**	61(18–82)
**Gender**	
Male	294(55.6%)
Female	235(44.4%)
**COPD**	14(2.6%)
**DM**	94(17.8%)
**HBP**	151(28.5%)
**PS**	
0–1	501(94.7%)
2–3	28(5.3%)
**ASA**	
1	52(9.8%)
2	436(82.4%)
3	40(7.6%)
4	1(0.2%)
**History of abdominal surgery**	108(20.4%)
**CAD**	26(4.9%)
**Cerebral vessel disease**	23(4.3%)
**Previous chemotherapy or radiotherapy**	20(3.8%)
**Preoperative biliary drainage**	157(29.7%)
**Preoperative hyperbilirubinemia**	246(46.5%)
**Weight loss**	290(54.8%)
**Smoking**	162(30.6%)
**Alcohol abuse**	105(19.8%)

*COPD* chronic obstructive pulmonary disease, *HBP* hypertension, *DM* diabetes mellitus, *PS* performance status, *CAD* coronary artery disease, *ASA* American Society of Anaesthesiologists

**Table 2:** Intraoperative variables and postoperative complications

Variables	Median (range)/number(%)
**Operation time (min)**	287(150–648)
**Intraoperative blood loss (ml)**	200(50–1500)
**Transfusion**	68(12.9%)
**RBC transfused(U)**	4(2–20)
**Pancreatic remnant anastomosis**	
PJ	150(28.4%)
PG	364(68.8%)
No anastomosis	15(2.8%)
**Combined PV-SMV or IVC resection**	20(3.8%)
**Operation types**	
PD	436(82.4%)
PD combined devisceration	78(14.7%)
TP	10(1.9%)
TP combined devisceration	5(0.9%)
**Morbidity**	311(58.8%)
**Clavien-Dindo classification**	
I	51(9.6%)
II	183(34.6%)
IIIa	56(10.6%)
IIIb	11(2.1%)
IVa	10(1.9%)
**DGE**	77(14.6%)
B	22(4.2%)
C	55(10.4%)
**PPH**	77(14.6%)
A	4(0.8%)
B	63(11.9%)
C	10(1.9%)
**Bile leakage**	21(4.0%)
**Pneumonia**	12(2.3%)
**Abdominal infection**	84(15.9%)
**Urinary tract infection**	3(0.6%)
**Wound infection**	5(0.9%)
**Arrhythmia**	10(1.9%)
**Postoperative pancreatic fistula**	204(38.6%)
A	152(28.7%)
B	46(8.7%)
C	6(1.1%)
**Percutaneous drainage**	53(10.0%)
**RBC transfusion**	124(23.4%)
**RBC transfused(U)**	4(2–30)
**Relaparotomy**	16(3.0%)
**Readmission**	6(1.1%)
**Postoperative hospital stay (days)**	19(8–121)

***PV-SMV*portal vein-superior mesenteric vein,*IVC*inferior vena cava,*PD*pancreaticoduodenectomy,*TP*total pancreatectomy,*DGE*delayed gastric emptying,*PPH*postpancreatectomy haemorrhage,*RBC*red blood cell**

### Postoperative complications

As shown in Table 2, postoperative complications according to the Clavien-Dindo classification system occurred in 311 patients (58.8%). POPF occurred in 204 patients (38.6%), with grade A POPF in 152 patients (28.7%), grade B POPF in 46 patients (8.7%), and grade C POPF in 6 patients (1.1%). POPF relating abdominal hemorrhage developed in 9 patients. Among of them, 3 patients underwent angiopraphy and embolization, 5 patients underwent reoperation and one patient received conservative treatment. Twenty-one patients (4.0%) experienced bile leakage after the operation. Abdominal infection occurred in 84 patients (15.9%) and 53 patients (10.0%) received percutaneous drainage. One hundred and twenty-four patients (23.4%) received a postoperative RBC transfusion, and the median number of transfused RBCs was 4 units (range: 2–30 units).

### PG versus PJ

The treatment groups were balanced in terms of most clinical factors and surgical details. However, the hard pancreas (PJ vs. PG, 88.0% vs. 15.4%, *p* < 0.001) and dilated pancreatic duct rates (PJ vs. PG, 65.3% vs. 13.5%, *p* < 0.001) were significantly different between the 2 groups (Table 3). The PJ group patients also had a significantly higher percentage of pancreatic ductal adenocarcinoma or chronic pancreatitis than the PG group (50.0% vs. 18.1%, *p* < 0.001). The incidence of POPF was 42.6% after PG and 32.7% after PJ (*p* = 0.037). Soft pancreas texture, a normal pancreatic duct size, obesity, longer operation time and pancreaticogastrostomy were significant factors affecting POPF, with estimated odds ratios of 3.191 (*p* < 0.001), 3.928 (*p* < 0.001), 1.976(*p* = 0.001), 1.635(*p* = 0.040) and 0.323(*p* = 0.001) respectively (Table 4). In the subgroup analysis according to pancreas texture, the CR-POPF of PJ was higher than that of PG significantly in patients with soft pancreas texture (*p* = 0.033). The operation time of PJ was shorter than that of PG significantly in patients with hard pancreas texture (*p* = 0.035) (Table 5). In the subgroup analysis according to pancreatic duct size, intraoperative blood loss and operation time of PG was less than that of PJ significantly in patients with normal main pancreatic duct (*p* < 0.05) (Table 6).

**Table 3:** Comparison of the perioperative variables of the PJ and PG groups

	PJ(150)	PG(364)	p
MPD diameter(> 3 mm)	98(65.3%)	49(13.5%)	**< 0.001**
Pancreatic texture (hard)	132(88.0%)	56(15.4%)	**< 0.001**
Gender (male)	89(59.3%)	196(53.8%)	0.255
Pathology (PDAC or CP)	75(50.0%)	66(18.1%)	**< 0.001**
BMI	23.5 ± 3.2	23.6 ± 3.3	0.628
ASA			0.329
1	10(6.7%)	40(11.0%)	
2	131(87.3%)	294(80.8%)	
3	8(6.0%)	29(8.0%)	
4	0	1(0.3%)	
COPD	3(2.0%)	11(3.0%)	0.767
DM	27(18.0%)	61(16.8%)	0.734
HBP	45(30.0%)	101(27.7%)	0.607
History of abdominal surgery	35(23.3%)	67(18.4%)	0.203
Preoperative biliary drainage	50(33.3%)	104(28.6%)	0.284
Preoperative hyperbilirubinemia	87(58.0%)	157(43.1%)	**0.002**
Operation time (min)	296.4 ± 58.9	288.7 ± 65.3	0.211
Intraoperative blood loss (ml)	276.0 ± 186.2	236.4 ± 169.8	**0.010**
Postoperative hospital stay (days)	21.6 ± 12.2	24.2 ± 14.4	0.056
Complications	78(52.0%)	229(62.9%)	**0.022**
POPF	49(32.7%)	155(42.6%)	**0.037**
Grade A POPF	32(21.3%)	120(33.0%)	**0.009**
Grade B/C POPF	17(11.3%)	35(9.6%)	0.557
Relaparotomy	1(0.7%)	15(4.1%)	**0.048**
DGE	19(12.7%)	58(15.9%)	0.345
PPH	23(15.3%)	51(14.0%)	0.698

*PJ* pancreaticojejunostomy, *PG* pancreaticogastrostomy, *MPD* main pancreatic duct, *PDAC* pancreatic ductal adenocarcinoma, *BMI* body mass index, *ASA* American Society of Anaesthesiologists, *COPD* chronic obstructive pulmonary disease, *DM* diabetes mellitus, *HBP* hypertension, *DGE* delayed gastric emptying, *PPH* postpancreatectomy haemorrhage, *CP* chronic pancreatitis, *POPF* Postoperative pancreatic fistula

**Table 4:** Logistic Regression Analysis of factors affecting POPF

Parameter	Univariate Analysis		***P***	Multivariate Analysis		***P***
	Odds Ratio	95% CI		Odds Ratio	95% CI	
**Gender**			0.153			
Female	1					
Male	0.771	0.539–1.102				
**Gland texture**			**< 0.001**			**< 0.001**
Hard	1			1		
Soft	2.787	1.877–4.138		3.191	1.695–6.008	
**Pathology**			**< 0.001**			0.15
PDAC or CP	1			1		
Others	2.635	1.704–4.077		1.605	0.842–3.058	
**pancreatic duct diameter**			**< 0.001**			**< 0.001**
> 3 mm	1			1		
≤ 3 mm	4.14	2.601–6.591		3.928	2.217–6.961	
**Intraoperative blood loss**			0.814			
≤ 400 ml	1					
> 400 ml	1.076	0.582–1.992				
**Age**			0.725			
< 70	1					
≥ 70	0.913	0.550–1.516				
**BMI**			**< 0.001**			**0.001**
< 25	1			1		
≥ 25	2.068	1.421–3.008		1.976	1.326–2.945	
**ASA score**			0.4			
1-2	1					
3-4	0.743	0.303–1.134				
**Preoperative jaundice**			0.608			
No	1					
Yes	0.912	0.640–1.299				
**Alcohol abuse**			0.482			
No	1					
Yes	1.17	0.755–1.813				
**Diabetes mellitus**			0.462			
No	1					
Yes	1.19	0.748–1.894				
**Previous abdominal surgery history**			0.913			
No	1					
Yes	0.976	0.626–1.521				
**Preoperative biliary decompression**			0.176			
No	1					
Yes	1.302	0.888–1.910				
**Operation time**			**0.028**			**0.04**
≤ 330 min	1			1		
> 330 ml	1.622	1.055–2.494		1.635	1.022–2.617	
**Intraoperative RBC transfusion**			0.273			
No	1					
Yes	1.345	0.791–2.286				
**Pancraticoenteric anastomosis method**			**0.037**			**0.001**
Pancreticojejunostomy (PJ)	1			1		
Pancreticogastrostomy (PG)	1.529	1.025–2.279		0.323	0.163–0.639	
**Combined with vascular resection**			0.232			
No	1					
Yes	0.531	0.188–1.498				

*PDAC* pancreatic ductal adenocarcinoma;CP: chronic pancreatitis, *BMI* body weight index, *ASA* American Society of Anaesthesiologists

**Table 5:** Subgroup analysis according to pancreas texture: PG vs PJ

	For hard pancreas texture			For soft pancreas texture		
	PJ(132pts)	PG(56pts)	*p*	PJ(18pts)	PG(308pts)	*p*
Operation time (min)	293.0 ± 56.1	312.6 ± 62.0	**0.035**	321.3 ± 74.1	284.4 ± 65.0	**0.021**
Intraoperative blood loss (ml)	270.8 ± 191.7	253.6 ± 151.9	0.550	313.9 ± 137.0	233.3 ± 172.9	0.053
POPF	28.8%	16.1%	0.066	61.1%	47.4%	0.258
CR-POPF	9.1%	1.8%	0.071	27.8%	11.0%	**0.033**
Intraoperative RBC transfusion	12.9%	12.5%	0.943	16.7%	11.7%	0.527
PPH	14.4%	16.1%	0.768	22.2%	13.6%	0.298
Postoperative RBC transfusion	19.7%	21.4%	0.787	50.0%	23.7%	**0.012**
Postoperative hospital stay (days)	20.1 ± 11.3	22.4 ± 10.2	0.207	32.5 ± 12.8	24.5 ± 15.0	**0.028**
Postoperative complications	48.5%	53.6%	0.524	77.8%	64.6%	0.315
Reoperation	0.0%	1.8%	0.298	5.6%	4.5%	0.582
Postoperative percutaneous drainage	6.1%	5.4%	0.851	16.7%	12.7%	0.714
Dilated main pancreaitc duct(>3 mm)	71.2%	39.3%	**< 0.001**	22.2%	8.8%	0.079
Readmission	1.5%	0.0%	1.000	0.0%	1.3%	1.000
Postoperative abdominal infection	9.1%	10.7%	0.729	27.8%	19.8%	0.413

*PG* pancreaticogastrostomy, *PJ* pancreaticojejunostomy, *POPF* postoperative pancreatic fistula, *CR-POPF* clinically relevant postoperative pancreatic fistula, *PPH* post-pancreatectomy hemorrhage, *RBC* red blood cell

**Table 6:** Subgroup analysis according to main pancreatic duct diameter: PG vs PJ

	For dilated main pancreatic duct(> 3 mm)			For normal main pancreatic duct(≤3 mm)		
	PJ(98pts)	PG(49pts)	*p*	PJ(52pts)	PG(315pts)	*p*
Operation time (min)	290.3 ± 58.3	300.5 ± 61.6	0.328	307.9 ± 59.0	286.9 ± 65.7	**0.031**
Intraoperative blood loss (ml)	264.3 ± 193.7	277.6 ± 160.1	0.680	298.1 ± 170.6	230.0 ± 170.6	**0.008**
POPF	20.4%	14.3%	0.366	55.8%	47.0%	0.240
CR-POPF	7.1%	2.0%	0.269	19.2%	10.8%	0.083
Intraoperative RBC transfusion	11.2%	12.2%	0.855	17.3%	11.7%	0.262
PPH	14.3%	10.2%	0.487	17.3%	14.6%	0.613
Postoperative RBC transfusion	17.3%	20.4%	0.651	34.6%	23.8%	0.097
Postoperative hospital stay (days)	18.4 ± 10.3	18.4 ± 6.9	0.985	27.8 ± 13.0	25.1 ± 15.1	0.228
Postoperative complications	39.8%	44.9%	0.554	75.0%	65.7%	0.187
Reoperation	0.0%	0.0%	–	1.9%	4.8%	0.711
Postoperative percutaneous drainage	5.1%	4.1%	1.000	11.5%	12.7%	0.815
Hard pancreatic gland texture	95.9%	44.9%	**< 0.001**	73.1%	10.8%	**< 0.001**
Readmission	1.5%	0.0%	1.000	0.0%	1.0%	1.000
Postoperative abdominal infection	2.0%	2.0%	1.000	21.2%	20.3%	0.890

*PG* pancreaticogastrostomy, *PJ* pancreaticojejunostomy, *POPF* postoperative pancreatic fistula, *CR-POPF* clinically relevant postoperative pancreatic fistula, *PPH* post-pancreatectomy hemorrhage, *RBC* red blood cell

## Discussion

The present study reported 529 consecutive PDs, representing the largest consecutive study of PD without mortality. Although the occurrence of pancreatic fistulas was 38.6%, routine evaluation of amylase level of the drainage fluid and intermittent irrigation through drainage tube might prevent POPF related mortality. Recent advances in surgical techniques and adequate management of postoperative complications have led to improved clinical outcomes of PD, and the mortality following PD has decreased to below 6% [8]. POPF is a main source of major morbidity due to the intraperitoneal release of enterokinase and activation of pancreatic proenzymes resulting in sepsis and haemorrhage. This complication might be inevitable and still causes troublesome short-term outcomes after surgery.

The approach to management PA remains key factor in determining the chance of developing a POPF. Despite multiple randomized studies and meta-analyses, there is no clear evidence or universally accepted guidelines for how to construct the optimal PA after PD. [9] The multiple studies described above have failed to provide definitive, consistent, and convincing level 1 evidence that any one technique of PA is better than the others, either during the traditional open PD or more recently with the laparoscopic PD. Therefore, it should be expected to utilize different forms of PA depending on pancreatic texture and main pancreatic duct in selected situations, which might be a potential solution to evade problem of POPF. PJ is the commonly preferred anastomosis method. Many techniques have been proposed for the reconstruction of pancreatic digestive continuity to prevent complications after PD. [10–12] PG anastomosis has an excellent blood supply, less tension in the anastomosis, and a thick stomach wall, which facilitate the establishment of a sound anastomosis [13]. Furthermore, the acid milieu of the stomach and the absence of enterokinase protect the anastomosis from autodigestion by inactivating the pancreatic proenzymes [14]. Previous studies reported contradictory results regarding the impact of PG versus PJ on the postoperative fistula rate [15–17]. Recently, reconstruction by PG was associated with lower postoperative pancreatic and biliary fistula rates [18]. These principles include good exposure and visualization, the use of a fine, nonstrangulating suture to produce a water-tight patent anastomosis, preservation of the blood supply, tension-free fixation of the gastrointestinal tract to the pancreas, and coverage of the transected pancreas [19]. The present study identified the morbidity of GJ was higher than that of PG significantly for patients with soft pancreas. Generally, it recommended that PG was an optimal approach for these patients.

This study contained 52 patients (9.8%) with clinically relevant PF after PD, which was highly consistent with previous studies. A grade A POPF was not considered clinically important; thus, only grade B/C should account for the incidence of clinically relevant POPF [20]. The prevention of clinically relevant POPF may partially depend on the prompt healing of pancreaticodigestive tract anastomoses, which is attributed to the immediate recovery from a minor pancreatic fistula originating from the pancreatic branch duct or parenchyma at the pancreas surface [5]. Prophylactic drains after pancreatic surgery allow physicians to monitor the occurrence of intra-abdominal bleeding and to detect and drain a pancreatic, biliary, or enteric fistula [21]. Intermittent irrigation aimed to dilute the concentration of intra-abdominal amylase level, which is effective in preventing damage caused by the erosive retention of pancreatic secretions. For symptomatic abdominal collection fluid or abdominal abscess, percutaneous puncture and drainage was the preferred procedure. The reported success rate of the conservative treatment of a POPF is approximately 80%. The relaparotomy should be performed only when patients presented a high output fistula and severe sepsis or haemorrhage and cannot be managed by other means [22].

The results were consistent with the literature that reported a significantly elevated risk of post-PD bleeding in patients with pancreatic fistulas [23, 24]. It was also confirmed that pancreatic leakage and intra-abdominal abscess were correlated to post-PD bleeding [25]. Therefore, any procedure that can prevent pancreatic fistula or intra-abdominal abscess can decrease the post-PD bleeding rate. Prophylactic irrigation around a PJ was reported to possibly decrease the incidence of pancreatic fistulas and infectious complications [26]. It was routinely performed a low-speed intermittent irrigation was added when the drain fluid turned turbid with sediment.

## Conclusions

Individualized pancreaticoenteric anastomosis should be determined based on the pancreatic texture and diameter of the main pancreatic duct. The appropriate anastomosis and postoperative management could prevent mortality.

## Supplementary information

**Additional file 1.**

## Data Availability

The datasets generated and/or analyzed during the current study are not publicly available due to protecting individual patient privacy but are available from the corresponding author on reasonable request.

## Abbreviations

CR-POPF: Clinically relevant postoperative pancreatic fistula
PG: Pancreaticogastrostomy
PJ: Pancreaticojejunostomy
BMI: Body mass index
PD: Pancreaticoduodenectomy
POPF: Postoperative pancreatic fistula
PA: Pancreaticoenteric anastomosis
ASA: American Society of Anaesthesiologists
