# Supplementary material for: The individualized selection of Pancreaticoenteric anastomosis in Pancreaticoduodenectomy
Source: BMC Surg. 2020 Jun 22;20:140. doi: 10.1186/s12893-020-00791-y (PMC7310108; doi:10.1186/s12893-020-00791-y)
Supplement: Supplementary file 1 — Additional file 1. [file 12893_2020_791_MOESM1_ESM.docx]

| **Supplementary Table 1: Primary tumor pathological diagnosis** | | |
| --- | --- | --- |
| **primary tumor location** | **Benign(52)** | **Malignant(477)** |
| **pancreas** | **44** | **154** |
| **distal bile duct** | **0** | **87** |
| **Vater ampulla** | **0** | **92** |
| **duodenum** | **8** | **112** |
| **colon** | **0** | **22** |
| **others** | **0** | **10** |
